# Supplementary material for: Isolation and characterization of a pangolin-borne HKU4-related coronavirus that potentially infects human-DPP4-transgenic mice
Source: Nat Commun. 2024 Feb 5;15:1048. doi: 10.1038/s41467-024-45453-2 (PMC10844334; doi:10.1038/s41467-024-45453-2)
Supplement: Supplementary file 1 — Supplementary Information [file 41467_2024_45453_MOESM1_ESM.pdf]

Supplementary Information

Supplementary Table 1 | List of primers used in this study 1
Supplementary Table 2 | ORF1ab gene probe sequences for RNA FISH 2
Supplementary Fig. 1 | Analyses of pangolin coronavirus HKU4-associated virus sequences 3
Supplementary Table 3 | High-throughput sequencing results of the pangolin-CoV-HKU4-P251T cultures of each passage 5
Supplementary Fig. 2 | Virus infection in ChaGo-K-1 and Tb 1 Lu cells 6
Supplementary Fig. 3 | Experimental infection of pangolin-CoV-HKU4-P251T in hDPP4-transgenic mice 7
Supplementary Table 4 | Neutralization antibody titers of mice serum against pangolin-CoV-HKU4-P251T 9
Supplementary Fig. 4 | Pathology observed in lung of virus-infected WT-mice 10
Supplementary Fig. 5 | Immunohistochemical analysis of T and B lymphocytes in lung from virus-infected hDPP4- and WT-mice 11

Supplementary Table 1 | List of primers used in this study

**Gene**	**Primer**	**Sequence** (5′ to 3′)	**Reference**
ORF1ab of PCoV-HKU4-P251T	P251T_qF1	GGTTTCAAGCGTCAGATTAG	This study
	P251T_qR1	AGCACCACCAACGATTTTAG	
E sgmRNA of PCoV-HKU4-P251T	P251E_sgF	GAACTTTGATTTTAACGAAC	This study
	P251E_sgR	GTAAGGATTGCTAAACACAC	
Mouse β-actin	Mus_Actb_F	GCAGGAGTACGATGAGTCCG	This study
	Mus_Actb_R	ACGCAGCTCAGTAACAGTCC	
IFN-β	LPW34090	GCGGACTTCAAGATCCCTATG	(Lau et al. 2021)
	LPW34091	ACAATAGTCTCATTCCACCCAG	
IFN-γ	LPW34092	AAATCCTGCAGAGCCAGATTAT	
	LPW34093	GCTGTTGCTGAAGAAGGTAGTA	
Mx1	LPW36687	GGGGAGGAAATAGAGAAAATGAT	
	LPW36688	GTTTACAAAGGGCTTGCTTGCT	
IL-2	LPW34070	GGACTTTCTGAGGAGATGGATAG	
	PW34071	TGTTGTAAGCAGGAGGTACATAG	
IL-12p40	LPW34074	TTGAACTGGCGTTGGAAGCACG	
	PW34075	CCACCTGTGAGTTCTTCAAAGGC	
TNF-α	LPW34076	TTGTCTACTCCCAGGTTCTCT	
	PW34077	GAGGTTGACTTTCTCCTGGTATG	
CXCL-1(KC)	LPW34084	TCCAGAGCTTGAAGGTGTTGCC	
	LPW34085	AACCAAGGGAGCTTCAGGGTCA	
G-CSF	PW34078	GCAGGCTCTATCGGGTATTTC	
	LPW34079	CACCCCTAGGTTTTCCATCTG	

Supplementary Table 2 | ORF1ab gene probe sequences for RNA FISH

**PCoV-HKU4-P251T** (5′ to 3′)	
GTGACATGGTCACTTCTTTT	ATCTTCATTACTACGGGCAT
CATCTGTATCACAGAGTGGT	TCCACACCTTTACTACGTAA
CTCAAACCATGGTGTGAGTT	ACATACCCAAAAGGCATTGC
CACTAGGTTGAAGGCAGTTT	CGCTAAATCAAGTCCGTGTG
AGTGCTGAGAAAGCATACCT	AAGCAACTTCAGGTGTTTGC
AATGGTGTATTCACCAGTCA	AGATGTCAAATAAGCCCTGC
CTGGTGTAAAATCACCACCA	AACCAATGGCTGCAGTGAAT
AACCTTCCTTAGCTACTATT	AGTCTGCAAAGCAGGTACTA
ACACGAGATTGAACATCCGC	CTGGTACTATAAGTTCAGCC
ATAGCACCACAGTCACAAGA	CCACGCCTTGAAAGACATTA
CTATGGTTACAACTGCCATC	GTTTTAGTTAAGGTGCCAGA
ACCGTCTAAGACGCTGTAAG	TTTAGGGTACCATACACTGG
TACCATCACAACAACCCATG	CAGGCAATATAGTAGCTGGT
GCATCACCAATGCGTGAAAC	AGATGGGCTTTGTATCATCA
ATACCAGTTGCACCTATATT	TTACCATTATACATGGCACC
GAACCTGCCCATGAAATTTT	AATTGACGAAGACTAGCCCT
TTGTTATAATCGCCTGGCAA	TGCAAAGAGGGACGATGCAC
AACGTCAACAGTGTCTGCAT	CAACGCCAGCAGTTCTTAAA
ATAATTCCCTACAACCATGC	CCAGTCTACACGCTTAAACT
CAGCTTTACCATTAGTGCAA	CCAACAATCCTGTAACATCC
TGAATTCCATGCACAGTCAC	TCTTGTCCTATAAGACACCA
ACTCTACACTTGGTTCATGT	ATGGTAGAAGCCTCAACACG
AGGGTTAGCAATAGCTTTCG	GGCGATTTCTCTACTATCAC
TCCATGACCCTTAAGTAACA	AGCTTATCAAGCTTGTCACG

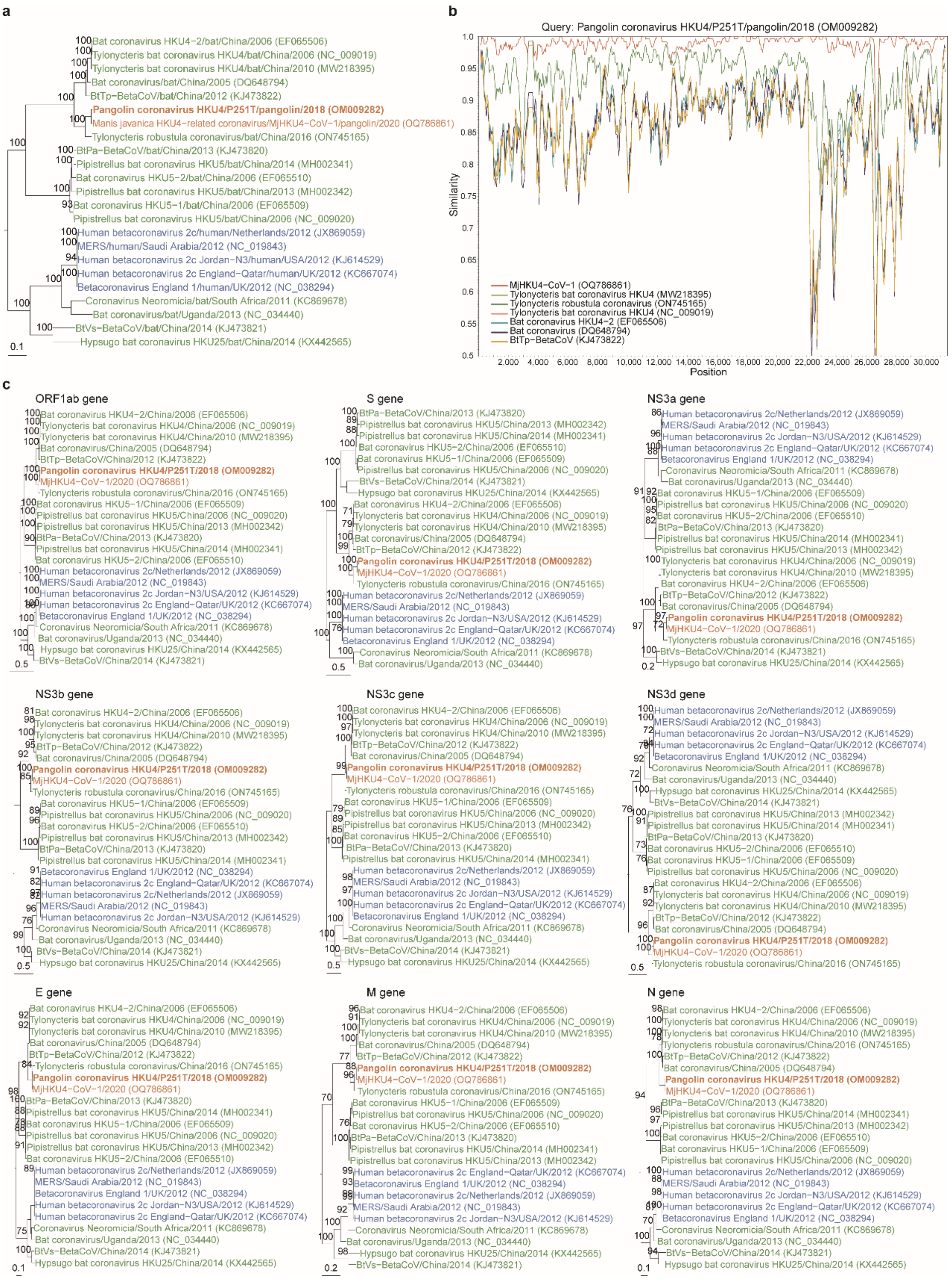

Supplementary Fig. 1 | Analyses of pangolin coronavirus HKU4-associated virus sequences. **a** and **c**, Phylogeny of the subgenus *Merbecovirus* estimated from the complete genomes (**a**), and phylogenetic trees of different genomic regions (**c**). Viral sequences obtained from pangolin, bat and human are shown in orange, green and blue label of node, respectively. Branch supports obtained from 1,000 bootstrap replicates are shown. The midpoint rooting algorithm were used to set the roots of all phylogenies. **b**, Recombination analysis. Sliding window analysis of changing patterns of sequence similarity using pangolin coronavirus HKU4/P251T (OM009282) against other viral sequences related

P251T in the branch. The similarities to different reference sequences are indicated by different colors. In the scanning, the sliding window size was set to 200 bp and the step size was 20 bp.

Supplementary Table 3 | High-throughput sequencing results of the pangolin-CoV-HKU4-P251T cultures of each passage

**Passage No.**	**Sample number**	**SRA accessions of sequencing data**
1	P251T/P1	SRR25655213
2	P251T/P2	SRR25655212
3	P251T/P3	SRR25655206
4	P251T/P4	SRR25655205
5	P251T/P5	SRR25655204
6	P251T/P6	SRR25655203
7	P251T/P7	SRR25655202
8	P251T/P8	SRR25655201
9	P251T/P9	SRR25655200
10	P251T/P10	SRR25655199
11	P251T/P11	SRR25655211
12	P251T/P12	SRR25655210
13	P251T/P13	SRR25655209
14	P251T/P14	SRR25655208
15	P251T/P15	SRR25655207

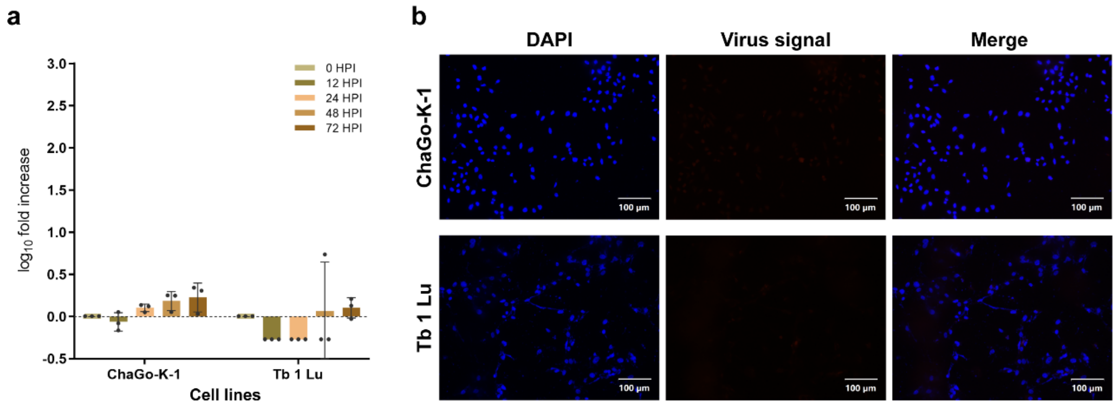

Supplementary Fig. 2 | Virus infection in ChaGo-K-1 and Tb 1 Lu cells, related to Fig. 2.
a, Kinetics of replication of pangolin-CoV-HKU4-P251T in ChaGo-K-1 and Tb 1 Lu cell lines. Viral RNA levels of virus in cell culture supernatant were detected at 0, 12, 24, 48, and 72 HPI, with three independent biological replicates per time point and three technical replicates per sample. The viral RNA levels were normalized relative to initial phase of infection (0 HPI). Data are presented as mean ± SD (shown as error bars). ANOVA was used for multiple comparisons. **b**, Fluorescence in situ hybridization locating ORF1ab gene of pangolin-CoV-HKU4-P251T in each cell lines at 48 HPI. Nuclei, DAPI (blue); ORF1ab probe, Quasar 570 (red). Each imaging experiment was independently performed at least three times with similar results, and representative images are shown. Original magnification ×200. Source data are provided as a Source Data file.

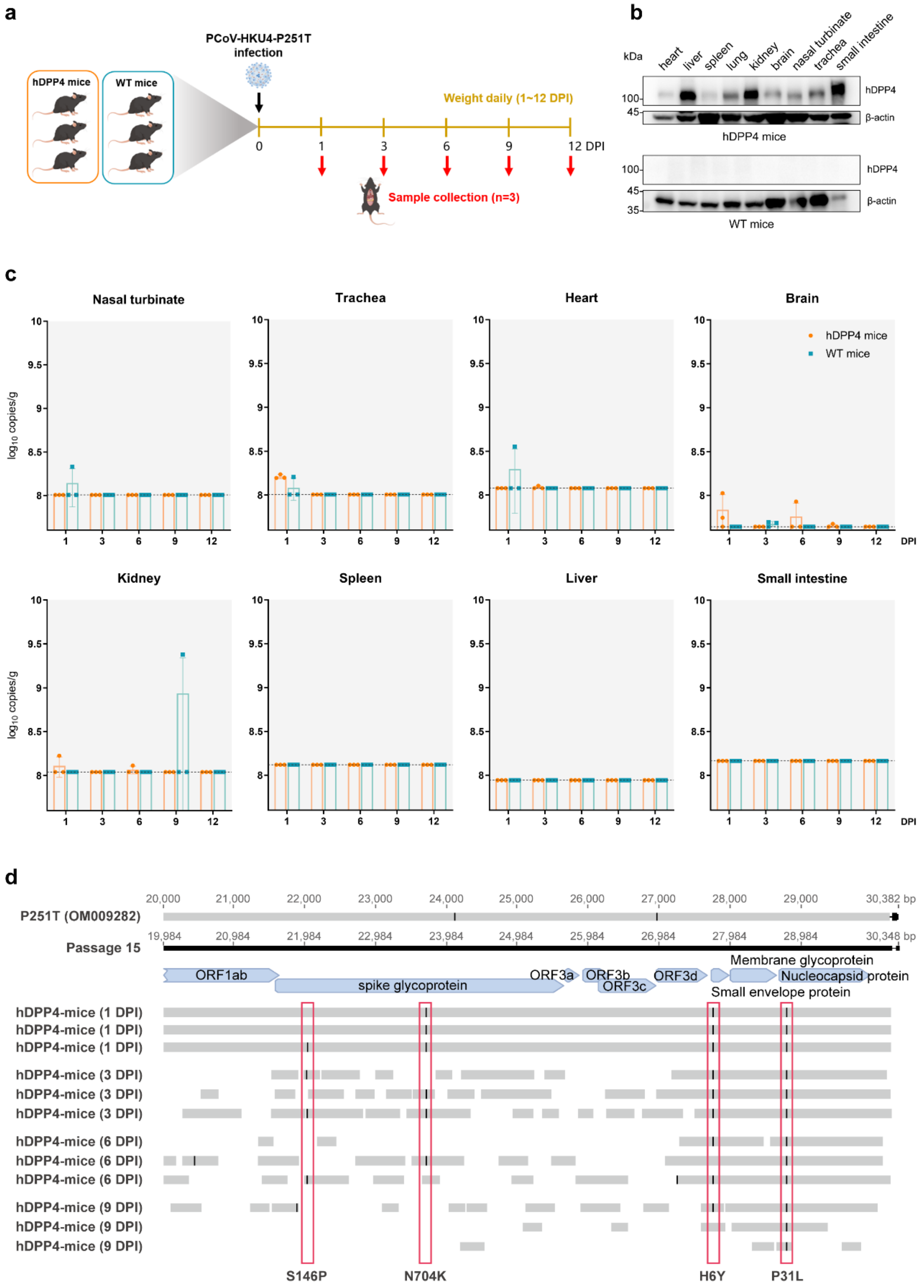

Supplementary Fig. 3 | Experimental infection of pangolin-CoV-HKU4-P251T in hDPP4-transgenic mice, related to Fig. 4.

a, Schematic of the experimental protocol. hDPP4-mice and WT-mice (5 weeks old) were infected intranasally with pangolin-CoV-HKU4-P251T in 50 µL of cell culture supernatant. An additional three hDPP4-mice and three WT-mice were mock-infected with an equal volume of cell culture supernatant to serve as controls. Bodyweight of the infected mice was monitored daily for 12 days (shown by

yellow line), and the survival of mice was recorded. At 1, 3, 6, 9, and 12 DPI, a subgroup of infected mice (n=3) was euthanized for sample collection (shown by red arrows). By Figdraw. **b**, Western blotting analysis of tissue distribution of hDPP4 in humanized mice and WT mice.Each imaging experiment was performed independently at least three times with similar results, and representative images of the results are shown. **c**, The viral RNA load in all organs other than lung of infected hDPP4 (orange) and WT (blue) mice. n=3 biologically independent animals per genotype for each time point. Data are presented as mean ± SD with scatter plot at each time point. Dashed lines denote the detection limit. Whenever the sample measurement was below the detection limit, the result was assigned a value equal to the minimum detection limit to facilitate statistical analysis of the data. **d**, Multiple amino acid sequence alignment of pangolin-CoV-HKU4-P251T from the lungs of hDPP4-mice. The sequence of the virus strain used for animal infection experiment (passage 15) was used as the reference sequence. Sequences names are noted on the left and the numbers in parentheses represent the number of days post-infection. The variant sites can be seen in the red boxes. Source data are provided as a Source Data file.

Supplementary Table 4 | Neutralization antibody titers of mice serum against pangolin-CoV-HKU4-P251T

Day post-infection	Mice No.	hDPP4-mice	WT-mice
1	1	<1:10	<1:10
	2	1:10	<1:10
	3	<1:10	<1:10
3	4	<1:10	<1:10
	5	<1:10	<1:10
	6	<1:10	<1:10
6	7	<1:10	<1:10
	8	1:20	<1:10
	9	1:10	<1:10
9	10	1:40	<1:10
	11	<1:10	<1:10
	12	1:10	<1:10
12	13	<1:10	<1:10
	14	<1:10	<1:10
	15	<1:10	<1:10

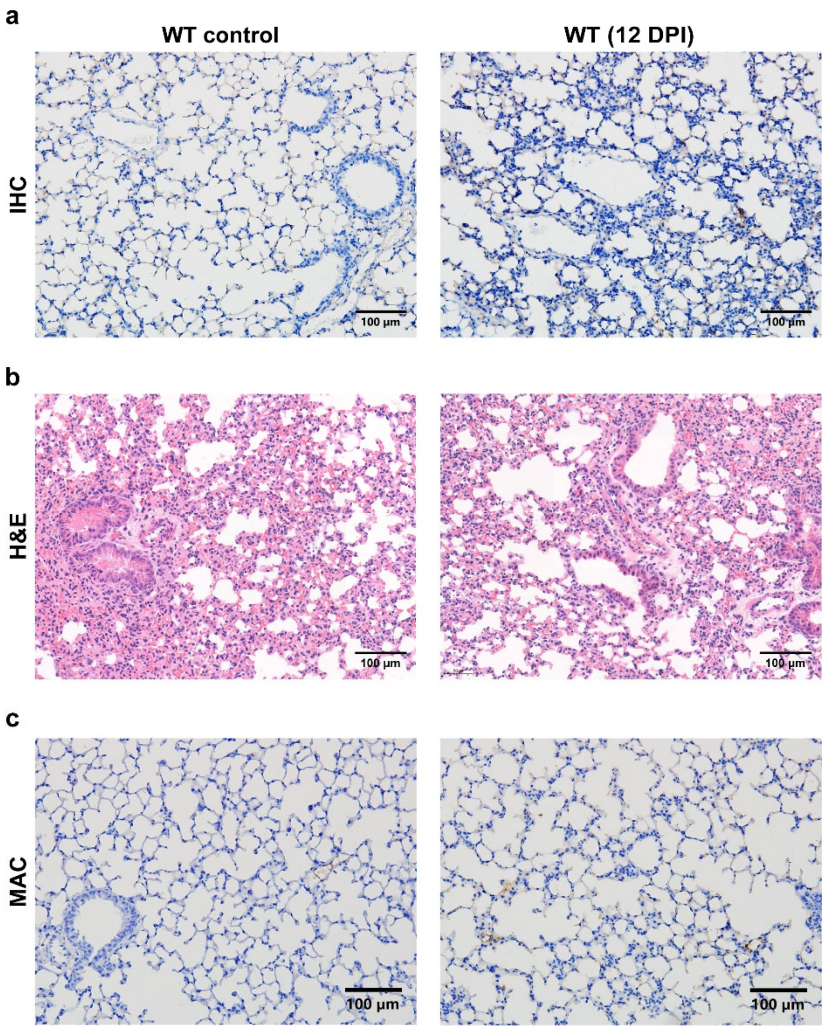

Supplementary Fig. 4 | Pathology observed in lung of virus-infected WT-mice.

a, Representative IHC staining images of viral nucleocapsid protein expression in lungs at 12 DPI of infected WT-mice. Related to Fig. 4b. **b**, Pathological features of virus-infected WT-mice. Related to Fig. 5b. **c**, Immunohistochemical analysis of lung tissue stained with MAC2 antibody for macrophage detection. Related to Fig. 5c. WT-mice inoculated with cell medium were used as control. Images are representative of three experimental animals. Original magnification ×200.

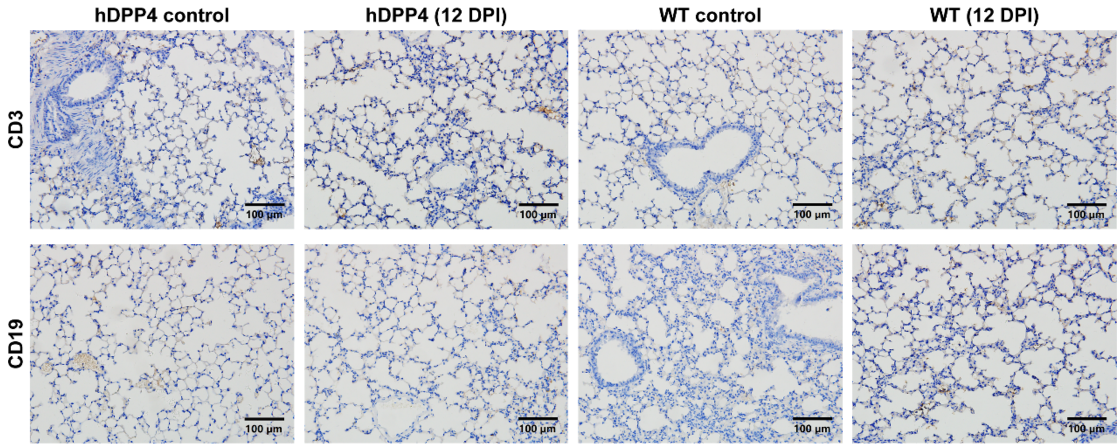

Supplementary Fig. 5 | Immunohistochemical analysis of T and B lymphocytes in lung from virus-infected hDPP4- and WT-mice.

IHC stained with CD3 antibody for T lymphocytes detection and CD19 antibody for B lymphocytes detection in lung of hDPP4- and WT-mice at 12 DPI. Mice inoculated with cell medium were used as control. Images are representative of three experimental animals. Original magnification ×200.
