# Supplementary material for: Isolation and characterization of a pangolin-borne HKU4-related coronavirus that potentially infects human-DPP4-transgenic mice
Source: Nat Commun. 2024 Feb 5;15:1048. doi: 10.1038/s41467-024-45453-2 (PMC10844334; doi:10.1038/s41467-024-45453-2)
Supplement: Supplementary file 3 — Reporting Summary [file 41467_2024_45453_MOESM3_ESM.pdf]

nature portfolio

Corresponding author(s): Jia-Fu Jiang, Yu-Wei Gao, and Wu-Chun Cao

Last updated by author(s): Dec 26, 2023

Reporting Summary

Nature Portfolio wishes to improve the reproducibility of the work that we publish. This form provides structure for consistency and transparency in reporting. For further information on Nature Portfolio policies, see our Editorial Policies and the Editorial Policy Checklist.

Statistics

For all statistical analyses, confirm that the following items are present in the figure legend, table legend, main text, or Methods section.

n/a	Confirmed	
☐	☒	The exact sample size (*n*) for each experimental group/condition, given as a discrete number and unit of measurement
☐	☒	A statement on whether measurements were taken from distinct samples or whether the same sample was measured repeatedly
☐	☒	The statistical test(s) used AND whether they are one- or two-sided<br>*Only common tests should be described solely by name; describe more complex techniques in the Methods section.*
☒	☐	A description of all covariates tested
☐	☒	A description of any assumptions or corrections, such as tests of normality and adjustment for multiple comparisons
☐	☒	A full description of the statistical parameters including central tendency (e.g. means) or other basic estimates (e.g. regression coefficient) AND variation (e.g. standard deviation) or associated estimates of uncertainty (e.g. confidence intervals)
☐	☒	For null hypothesis testing, the test statistic (e.g. *F*, *t*, *r*) with confidence intervals, effect sizes, degrees of freedom and *P* value noted<br>*Give P values as exact values whenever suitable.*
☒	☐	For Bayesian analysis, information on the choice of priors and Markov chain Monte Carlo settings
☒	☐	For hierarchical and complex designs, identification of the appropriate level for tests and full reporting of outcomes
☒	☐	Estimates of effect sizes (e.g. Cohen's *d*, Pearson's *r*), indicating how they were calculated

Our web collection on statistics for biologists contains articles on many of the points above.

Software and code

Policy information about availability of computer code

Data collection	No software was used.
Data analysis	AfterQC (v0.9.7), Trinity (v2.8.5), MAFFT (v7.505), TrimAl program, IQ-Tree (v2.2.0.3), ggtree package (v3.6.2), SimPlot (v3.5.1), GraphPad Prism (v7.0).

For manuscripts utilizing custom algorithms or software that are central to the research but not yet described in published literature, software must be made available to editors and reviewers. We strongly encourage code deposition in a community repository (e.g. GitHub). See the Nature Portfolio guidelines for submitting code & software for further information.

Data

Policy information about availability of data

All manuscripts must include a data availability statement. This statement should provide the following information, where applicable:

- Accession codes, unique identifiers, or web links for publicly available datasets
- A description of any restrictions on data availability
- For clinical datasets or third party data, please ensure that the statement adheres to our policy

High-throughput sequencing data have been deposited to Sequence Read Archive (SRR25655199-SRR25655213, Supplementary Table 3). All other relevant data are available within the article and Source Data file, or from the corresponding author upon request.

Research involving human participants, their data, or biological material

Policy information about studies with human participants or human data. See also policy information about sex, gender (identity/presentation), and sexual orientation and race, ethnicity and racism.

Reporting on sex and gender	This research does not involve human participants.
Reporting on race, ethnicity, or other socially relevant groupings	This research does not involve human participants.
Population characteristics	This research does not involve human participants.
Recruitment	This research does not involve human participants.
Ethics oversight	This research does not involve human participants.

Note that full information on the approval of the study protocol must also be provided in the manuscript.

Field-specific reporting

Please select the one below that is the best fit for your research. If you are not sure, read the appropriate sections before making your selection.

☒ Life sciences ☐ Behavioural & social sciences ☐ Ecological, evolutionary & environmental sciences

For a reference copy of the document with all sections, see nature.com/documents/nr-reporting-summary-flat.pdf

Life sciences study design

All studies must disclose on these points even when the disclosure is negative.

Sample size	No statistical method was used to predetermine the sample size. Sample sizes for cell and animal experiments were determined based on the field standards for sufficient reproducibility and according to the 3Rs of animal ethics regulation.
Data exclusions	No data were excluded from the analyses.
Replication	All image collection and immunoblot experiments were repeated at least three times and the representative images are shown. All experiments were performed in technical triplicates.
Randomization	All studies were performed after randomization.
Blinding	The investigators were blinded during the experiment, outcome assessment, and data analysis.

Reporting for specific materials, systems and methods

We require information from authors about some types of materials, experimental systems and methods used in many studies. Here, indicate whether each material, system or method listed is relevant to your study. If you are not sure if a list item applies to your research, read the appropriate section before selecting a response.

Materials & experimental systems

n/a	Involved in the study	
☐	☒	Antibodies
☐	☒	Eukaryotic cell lines
☒	☐	Palaeontology and archaeology
☐	☒	Animals and other organisms
☒	☐	Clinical data
☒	☐	Dual use research of concern
☒	☐	Plants

Methods

n/a	Involved in the study	
☒	☐	ChIP-seq
☒	☐	Flow cytometry
☒	☐	MRI-based neuroimaging

Antibodies

Antibodies used	Rabbit anti-pangolin-CoV-HKU4-P251T Nucleoprotein Pab (Sino Biological Inc., customized, 1:100 dilution), HA antibody (Biolegend, MMS-101P, IFA: 1:200 dilution, WB: 1:500 dilution), goat anti-mouse IgG Alexa Fluor 488 (Abcam, ab150117, 1:200 dilution), goat anti-rabbit IgG Alexa Fluor 594 (Abcam, ab150080, 1:200 dilution), Anti-DPP4 antibody (Abcam, ab215711, 1:1000 dilution), Anti-

ACE2 antibody (Abcam, ab108209, 1:1000 dilution), HRP-labeled Goat Anti-Rabbit IgG(H+L) (Beyotime, A0208, 1:1000 dilution), HRP-labeled Goat Anti-Mouse IgG (H+L) (Beyotime, A0216, 1:1000 dilution), MAC2 antibody (Cedarlane Laboratories, CL8942AP, 1:1000 dilution), CD3 antibody (Sino Biological Inc., 108567-T08, 1:500 dilution), CD19 antibody (Cell Signaling Technology, 3574, 1:50 dilution).

Validation

Rabbit anti-pangolin-CoV-HKU4-P251T Nucleoprotein Pab (rabbit antibody, Sino Biological Inc., customized, 1:100 dilution)

HA antibody (mouse antibody, Biolegend, MMS-101P, IFA: 1:200 dilution, WB: 1:500 dilution)
https://www.biolegend.com/en-us/products/purified-anti-ha-11-epitope-tag-antibody-11374

goat anti-mouse IgG Alexa Fluor 488 (Abcam, ab150117, 1:200 dilution)
https://www.abcam.cn/products/secondary-antibodies/goat-mouse-igg-hl-alexa-fluor-488-preadsorbed-ab150117.html

goat anti-rabbit IgG Alexa Fluor 594 (Abcam, ab150080, 1:200 dilution)
https://www.abcam.cn/products/secondary-antibodies/goat-rabbit-igg-hl-alexa-fluor-594-ab150080.html

Anti-DPP4 antibody (rabbit antibody, Abcam, ab215711, 1:1000 dilution)
https://www.abcam.cn/products/primary-antibodies/dpp4-antibody-epr20819-ab215711.html

Anti-ACE2 antibody (rabbit antibody, Abcam, ab108209, 1:1000 dilution)
https://www.abcam.cn/products/primary-antibodies/ace2-antibody-epr4436-ab108209.html

HRP-labeled Goat Anti-Rabbit IgG(H+L) (Beyotime, A0208, 1:1000 dilution)
https://www.beyotime.com/product/A0208.htm

HRP-labeled Goat Anti-Mouse IgG (H+L) (Beyotime, A0216, 1:1000 dilution)
https://www.beyotime.com/product/A0216.htm

MAC2 antibody (rat antibody, Cedarlane Laboratories, CL8942AP, 1:1000 dilution)
https://www.cedarlanelabs.com/Products/Detail/CL8942AP?lob=AllProducts

CD3 antibody (rabbit antibody, Sino Biological Inc., 108567-T08, 1:500 dilution)
https://cn.sinobiological.com/antibodies/mouse-cd3-epsilon-cd3e-108567-t08

CD19 antibody (rabbit antibody, Cell Signaling Technology, 3574, 1:50 dilution)
https://www.cellsignal.cn/products/primary-antibodies/cd19-antibody/3574

Eukaryotic cell lines

Policy information about cell lines and Sex and Gender in Research

Cell line source(s)

Vero 81 (ATCC, Cat#CCL-81), Huh7 (Laboratory of Wu-Chun Cao), Caco-2 (Nanjing Cobioer Biosciences Co., Ltd., Cat#CBP60025), Calu-3 (Nanjing Cobioer Biosciences Co., Ltd., Cat#CBP60086), BEAS-2B (Nanjing Cobioer Biosciences Co., Ltd., Cat#CBP60577), ChaGo-K-1 (Nanjing Cobioer Biosciences Co., Ltd., Cat#CBP60181), Tb 1 Lu (CCTCC, Cat#GDC0190), HeLa (Laboratory of Yu-Wei Gao).

Authentication

Cells were not subjected to additional cell authentication.

Mycoplasma contamination

All cell lines tested negative for mycoplasma.

Commonly misidentified lines
(See ICLAC register)

None of the cell lines used in this study are listed as commonly misidentified.

Animals and other research organisms

Policy information about studies involving animals; ARRIVE guidelines recommended for reporting animal research, and Sex and Gender in Research

Laboratory animals

hDPP4-transgenic mice (C57BL/6-Dpp4, Shanghai Model Organisms Center, Inc., NM-HU-190042) and wild-type C57BL/6 (Beijing Weitong Lihua Biotechnology Co., Ltd.) were used in this study. Female, 5 weeks old. The housing environment included a 12-hours light / dark cycle with constant room temperature (22-24°C), humidity (45–65%), and free access to water and diet.

Wild animals

No wild animals were involved in this study.

Reporting on sex

Female mice were used on the basis of similar research reported in the literature.

Field-collected samples

The study did not involve samples collected from the field.

Ethics oversight

Animals Experimental Committee, and Ethics Committee of Changchun Veterinary Research Institute, approval number IACUC of AMMS-11-2022-011.

Note that full information on the approval of the study protocol must also be provided in the manuscript.

Plants

Seed stocks	This research does not involve plants.
Novel plant genotypes	This research does not involve plants.
Authentication	This research does not involve plants.
