# Supplementary figures and images for: Isolation and characterization of a pangolin-borne HKU4-related coronavirus that potentially infects human-DPP4-transgenic mice
Source: Nat Commun. 2024 Feb 5;15:1048. doi: 10.1038/s41467-024-45453-2 (PMC10844334; doi:10.1038/s41467-024-45453-2)

Figure 2a

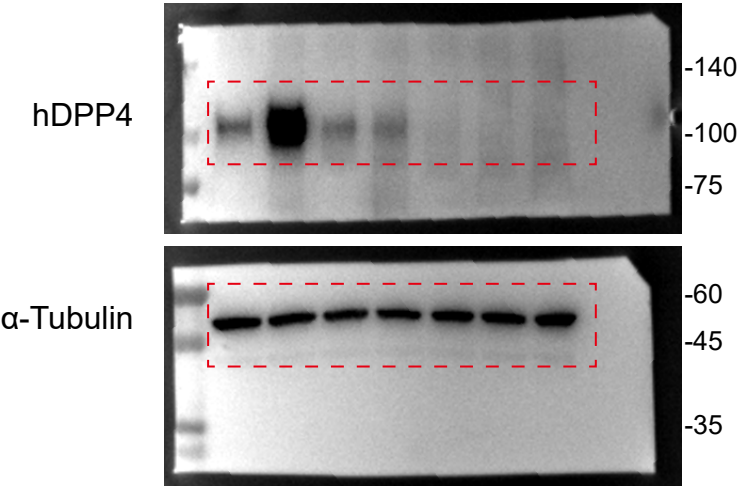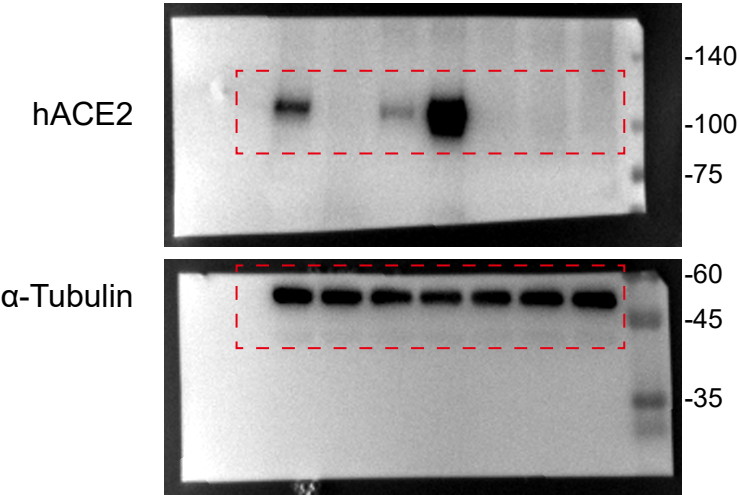

**Figure 3a and 3c**

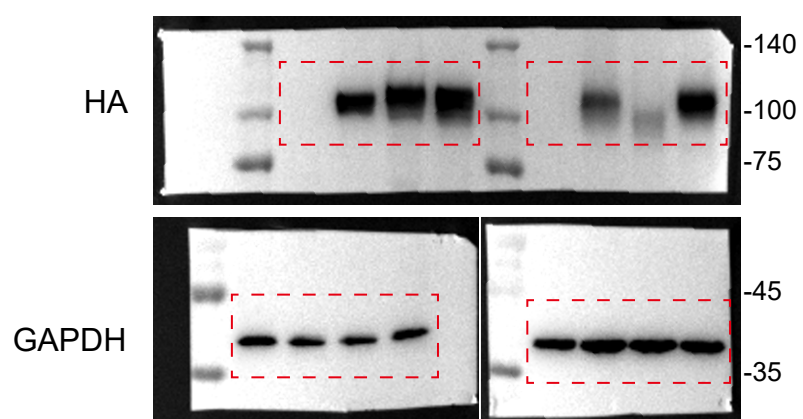

Figure S3b

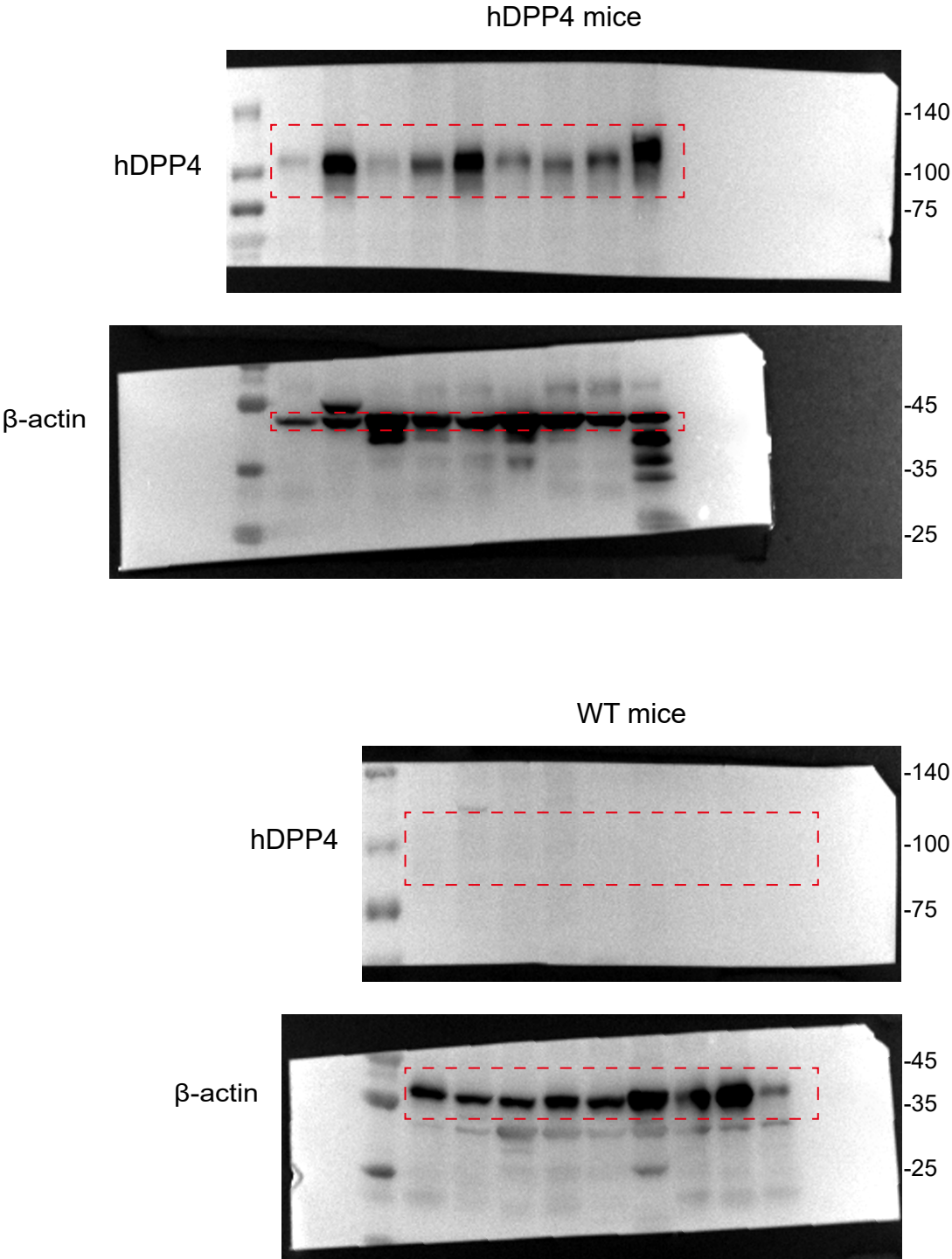

Supplement: Supplementary file 4 — Source Data [file 41467_2024_45453_MOESM4_ESM.zip › source data .pdf]
